# Two new species of harvestmen (Opiliones, Eupnoi, Neopilionidae) from Waitomo, New Zealand

**DOI:** 10.3897/zookeys.434.7486

**Published:** 2014-08-14

**Authors:** Christopher K. Taylor, Anna Probert

**Affiliations:** 1 Dept of Environment and Agriculture, Curtin University, GPO Box U1987, Perth, WA 6845, Australia; 2 School of Biological Sciences, University of Auckland, Private Bag 92019, Auckland Mail Centre, Auckland 1142, New Zealand

**Keywords:** Taxonomy, arachnids, cave biota

## Abstract

Two new species of harvestman (Opiliones: Neopilionidae: Enantiobuninae) are described from the Waitomo region of the North Island, New Zealand, *Forsteropsalis bona* **sp. n.** and *F. photophaga* **sp. n.** Both have been collected within caves in the region, where predation on glow-worms *Arachnocampa luminosa* has been previously recorded for one or both species (misidentified as ‘*Megalopsalis tumida*’). However, both are regarded as troglophiles rather than strict troglobites due to the presence of specimens outside the cave systems, and the absence of troglobitic adaptations. *Megalopsalis tumida* (Forster, 1944) is identified as a junior synonym of *Forsteropsalis fabulosa* (Phillipps & Grimmett, 1932).

## Introduction

The Waitomo region of the North Island, New Zealand, has become internationally renowned as a tourist attraction owing to its extensive cave systems. The primary reason for their fame is their large population of glow-worms *Arachnocampa luminosa* (Keroplatidae). These luminescent fly larvae construct silken nests on the roof of the cave from which they hang sticky threads to capture flying insects attracted to their light (Richards 1960). They are themselves predated upon by harvestmen (Opiliones), which are able to avoid entanglement by the glow-worms’ threads and pull the larvae from their nests (Richards 1960; Meyer-Rochow and Liddle 1988).

Richards (1960) recorded two species of harvestmen feeding on glow-worms at Waitomo. One, *Hendea myersi cavernicola* Forster, 1954 (Triaenonychidae), was originally described from Waitomo. The second species was identified by both Richards (1960) and Meyer-Rochow and Liddle (1988) as *Megalopsalis tumida* (Forster, 1944) (Neopilionidae). Richards (1960) recorded this species feeding only on mature glow-worm gnats; Meyer-Rochow and Liddle (1988) recorded it also feeding on pupae and late-instar larvae. *Megalopsalis tumida* is a junior synonym of *Forsteropsalis fabulosa* (Phillipps & Grimmett, 1932) (see below), and was originally described from near Wellington. Examination of specimens collected from caves in the Waitomo region revealed the presence of two species of Neopilionidae, both of them described as new below. Which of these was the species mentioned by Richards (1960) and Meyer-Rochow and Liddle (1988) is unknown. *Forsteropsalis bona* sp. n. is the more similar to *Forsteropsalis fabulosa*, but a photograph of ‘*Megalopsalis tumida*’ in Meyer-Rochow and Liddle (1988) may show *Forsteropsalis photophaga* sp. n. It is not impossible that the two were confused.

While Meyer-Rochow and Liddle (1988) regarded *Hendea myersi cavernicola* as a true troglobite, and did not find it outside the cave entrance, the collection of small numbers of ‘*Megalopsalis tumida*’ outside the cave led them to regard it as troglophilic rather than troglobitic. Similar habits were inferred by Taylor (2013) for *Megalopsalis suffugiens* Taylor, 2013, described from caves in Western Australia. Further epigean specimens are recorded herein for *Forsteropsalis bona*, while specimens collected in the cave were only a short distance from the entrance. *Forsteropsalis photophaga* has not yet been conclusively recorded outside the caves, but troglophily is also suggested for this species by the absence of strong adaptations for troglobitism.

## Methods

Specimens were sourced from the collection of Te Papa Tongarewa, Wellington, New Zealand (MONZ) or collected by hand by A. Probert and associates. Specimens collected by A. Probert will be deposited at the New Zealand Arthropod Collection, Landcare Research, Auckland, New Zealand (NZAC). All specimens are assigned to the area code WO (Waikato) by the system established by Crosby et al. (1998). Photographs and measurements were taken using a Nikon SMZ1500 stereo microscope and the NIS-Elements D 4.00.03 programme, and a Leica DM2500 compound microscope. Measurements are given in millimetres (mm). Coloration is described as in alcohol (live coloration is given in parentheses).

## Taxonomic descriptions

### Forsteropsalis bona sp. n.

Taxon classificationAnimaliaOpilionesNeopilionidae

http://zoobank.org/32F7E0A3-1CE0-41EF-B7AA-48DDAE2A529A

Figure 1

#### Holotype male.

**WO.** Lucky Strike Cave, Te Kuiti, on wall near entrance, 14 Feb 1959, K. A. J. Wise (MONZ).

#### Paratypes.

**WO.** 2 males, Waitomo Valley, in stream crevice outside cave entrance (shady), specimens intertwined and sluggish, 30 Mar 1959, L. G. Watson (MONZ); 1 male, Weir Cave, Stubbs Farm, Waitomo, ca. 2 m from cave entrance within a 15 cm radius of *Arachnocampa luminosa* larvae, 23 Aug 2010, A. Probert (NZAC).

#### Etymology.

From the Latin *bonus*, good, in contrast to the related *Forsteropsalis fabulosa*.

#### Male (n=4).

**Figure 1.**
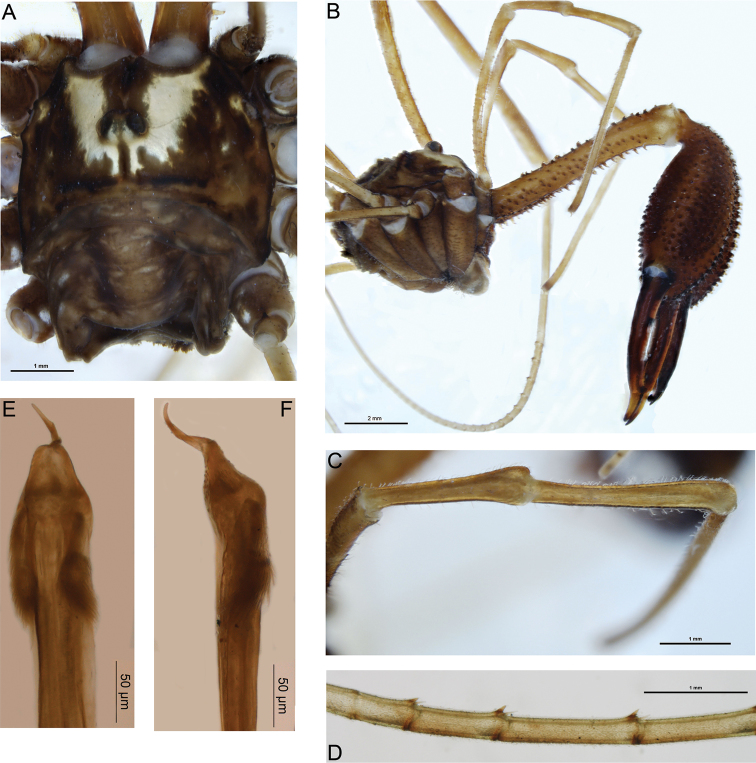
*Forsteropsalis bona* sp. n. **A** dorsal view of body, holotype **B** lateral view of body, pedipalps and chelicerae, holotype **C** dorsal view of right pedipalpal patella and tibia, holotype **D** proximal pseudosegments of right distitarsus I (venter upwards), holotype, showing ventrodistal teeth **E** penis, ventral view, specimen from Waitomo Valley **F** penis, right lateral view, specimen from Waitomo Valley.

Total body length 4.8–6.6; prosoma length 2.5–2.8, width 4.0–4.2. Prosoma (including ocularium) unarmed (Fig. 1A); ground colour in alcohol orange-brown with longitudinal yellow stripes on either side of ocularium (live coloration very dark brown [almost black] with orange-yellow stripes; appendages also black). Ozopores elongate, with small flanking lobes. Opisthosoma grey-yellow. Mouthparts cream-coloured; medial side of pedipalpal coxa with dense array of sharp denticles; cervix unarmed. Coxae yellow. *Chelicerae* (Fig. 1B): Segment I length 6.2–8.4; segment II 9.4–10.4. Elongate; segment I orange with lighter yellow patch at distal end, segment II dark orange-brown. Segment I denticulate, with denticles concentrated along dorsal, proventral and retroventral margins. Segment II massively inflated, evenly denticulate. Cheliceral fingers elongate, widely bowed apart; setae present on distal half of mobile finger. *Pedipalps*: Femur length 5.4–5.7; patella 2.2–2.4; tibia 2.8–3.1; tarsus 5.8–6.5. Distinctly elongate, yellow. Femur dorsally denticulate on proximal two-thirds; remainder of pedipalp unarmed. Setae sparse except for small concentration at prodistal end of patella; microtrichia present on tarsus and distal half of tibia; prodorsal end of patella with distinct protrusion but without definite finger-like apophysis (Fig. 1C). Tarsal claw without ventral tooth-row. *Legs*: Legs I femur length 8.6–9.9, patella 1.9–2.5, tibia 8.3–9.8; leg II femur 14.3–17.2, patella 2.2–2.8, tibia 14.4–17.8; leg III femur 7.5–8.7, patella 1.7–2.3, tibia 5.3–8.2; leg IV femur 8.5–10.7, patella 1.8–2.7, tibia 10.2–10.8. Femora sparsely denticulate, particularly in proximal half; remainder of legs unarmed. Distitarsus I with strong ventral tooth at distal end of each of first five or six pseudosegments (Fig. 1D). Tibia II with nine to fifteen pseudosegments; tibia IV with two pseudosegments. *Penis* (Fig. 1E–F): Shaft subquadrate; tendon long. Bristle groups relatively long, posterior bristle group with longest bristles reaching dorsal margin in lateral view. Glans short, subtriangular in ventral view, narrowing rapidly in lateral view.

#### Comments.

Females of this species are currently unknown. *Forsteropsalis bona* can be distinguished from most other *Forsteropsalis* species by its unarmed prosoma and enormous, sub-globose cheliceral segment II with widely bowed cheliceral fingers (Taylor 2011). In these features it strongly resembles *Forsteropsalis fabulosa*, and would key out to either *Forsteropsalis fabulosa* or *Forsteropsalis tumida* in the key to *Forsteropsalis* species provided by Taylor (2011). These two species are synonymised below. *Forsteropsalis bona* can be distinguished from *Forsteropsalis fabulosa* by the form of the pedipalpal patella: *Forsteropsalis fabulosa* has a distinct finger-like prodistal apophysis on the patella (Phillipps and Grimmett 1932: Fig. C p. 732), while the patellar apophysis is almost absent in *Forsteropsalis bona* (Fig. 1C). *Forsteropsalis fabulosa* also has denticles both dorsally and ventrally on the pedipalpal femur, while *Forsteropsalis bona* has denticles dorsally only.

An interesting feature of *Forsteropsalis bona* is the presence of a strong ventrodistal tooth on the end of each of the proximal pseudosegments of the distitarsus. This tooth sits between the two spinose setae generally present on each tarsal pseudosegment in all Enantiobuninae (Fig. 1D). Such a feature has not previously been recorded for this subfamily, though it is also present in *Forsteropsalis fabulosa* (specimens from MONZ, details given in Taylor 2011). This may represent a distinct synapomorphy of these two species.

The glans of both *Forsteropsalis fabulosa* (Taylor 2011) and *Forsteropsalis bona* is relatively short compared to other *Forsteropsalis* species, and converges in shape on that of the Australian genus *Megalopsalis* (Taylor 2011, 2013). Nevertheless, the remaining features of these two species support a direct relationship with other New Zealand species of *Pantopsalis* and *Forsteropsalis*, and with *Forsteropsalis* in particular. These features include dorsal papillae on the glans (Taylor 2011), setae on the mobile finger of the chelicera (absent in *Megalopsalis* except *Megalopsalis caeruleomontium*; Taylor 2011, 2013), and an array of denticles on the medial side of the pedipalpal coxa (Fig. 3A; Taylor 2011).

### Forsteropsalis fabulosa

Taxon classificationAnimaliaOpilionesNeopilionidae

(Phillipps & Grimmett, 1932)

Macropsalis fabulosa Phillipps & Grimmett, 1932: 731–733, fig. p. 732.
Megalopsalis fabulosa (Phillipps & Grimmett) – Forster 1944: 186–187, figs 10–11 (misidentification of *Forsteropsalis inconstans*).
Megalopsalis tumida Forster 1944: 188–189, figs 4–6 syn. n.
Forsteropsalis fabulosa (Phillipps & Grimmett) – Taylor 2011: 51, figs 99–101, 2012: 49.
Forsteropsalis tumida (Forster) – Taylor 2011: 60–61, figs 124–127.

#### Comments.

*Forsteropsalis fabulosa* and *Forsteropsalis tumida* were distinguished in Taylor (2011) solely by the degree of dilation of the second cheliceral segment, with the latter supposedly more inflated in *Forsteropsalis tumida* than in *Forsteropsalis fabulosa*. A number of species of *Forsteropsalis* and its sister genus *Pantopsalis* are now known to vary in cheliceral dilation (Taylor 2004, 2011, 2013, and see *Forsteropsalis photophaga* below). *Forsteropsalis fabulosa* and *Forsteropsalis tumida* were both described from the Wellington district, and there seems to no longer be any justification for separating them as different species. *Forsteropsalis tumida* is therefore regarded herein as a junior synonym of *Forsteropsalis fabulosa* (syn. n.).

### Forsteropsalis photophaga sp. n.

Taxon classificationAnimaliaOpilionesNeopilionidae

http://zoobank.org/633102AB-D0FD-4F91-8A38-CFDF1ECDF08F

Figure 2

#### Holotype male.

**WO.** Waitomo, Gardners Gut Cave System, 200 yards from Zweihöllen entrance, 25 June 1977, W. L. Blundell (MONZ).

#### Paratypes.

**WO.** 1 male, Giants Cavern, Hollow Hill Cave, Te Kuiti, in ‘Crows Nest’, 60–70 ft high, 12 January 1958, coll. R. W. Taylor (MONZ); 1 male, Aussie Cave, Taumatamaire Rd, Waitomo County, 50 ft, 16 May 1966, K. A. J. Wise (MONZ); 2 males, Stubbs Farm, Waitomo, on rocky cave substrate, February 2013, G. Holwell et al. (NZAC); 2 males, Mangapohue Cave, Stubbs Farm, Waitomo, on rocky cave substrate, 21 Oct 2013, A. Probert & D. Townsend (NZAC).

#### Etymology.

From the Greek *phos*, light, and *phagein*, to eat, in reference to this species’ predation of the glow-worm *Arachnocampa luminosa*.

#### Male (n=7).

**Figure 2.**
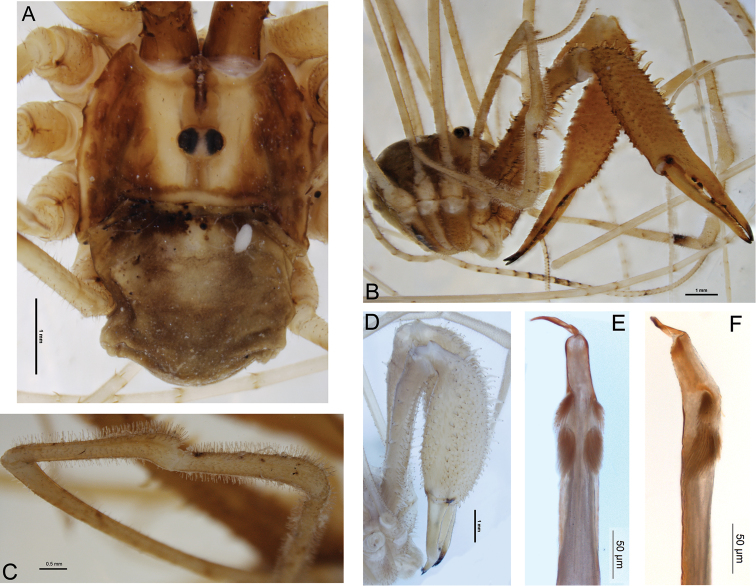
*Forsteropsalis photophaga* sp. n. **A** dorsal view of body, holotype, with parasitic mite attached **B** anterolateral view of body, pedipalps and chelicerae, holotype **C** dorsal view of right pedipalpal patella and tibia, holotype **D** anterolateral view of chelicerae of specimen from Aussie Cave, showing more inflated chelicerae **E** penis, ventral view, holotype **F** penis, right lateral view, holotype.

Total body length 3.5–6.1; prosoma length 1.9–2.1, width 2.5–3.9. Prosoma (including ocularium) unarmed except for few black setae (Fig. 2A); ground colour orange-brown with longitudinal yellow stripes on either side of ocularium (live colouration light to mid-brown with pale yellow stripes). Ozopores elongate, with small flanking lobes. Opisthosoma grey-brown. Mouthparts cream-coloured; medial side of pedipalpal coxa with array of sharp denticles; cervix with single pair of denticles laterally. Coxa I orange; remaining coxae and venter of opisthosoma yellow. *Chelicerae* (Fig. 2B, D): Segment I length 3.4–6.5, segment II 4.9–9.1. Elongate; orange except for lighter yellow patch at distal end of first segment. First segment dorsally with scattered denticles, becoming more elongate retrolaterally, ventrally with longitudinal prolateral and retrolateral rows of elongate denticles and some scattered median denticles proximally. Second segment mildly to notably inflated, sub-conical, evenly denticulate with longitudinal rows of more elongate denticles dorsally and retrolaterally. Cheliceral fingers elongate, slightly bowed apart; setae present along central third of mobile finger. *Pedipalps*: Femur length 4.6–6.5, patella 2.8–3.2, tibia 2.2–2.8, tarsus 4.8–5.7. Distinctly elongate; yellow. Median side of coxa with array of sharp denticles. Femur with few denticles dorsally in proximal half; remainder of pedipalp unarmed. Patella, tibia and proximal half of tarsus densely covered with plumose setae; microtrichia present over entirety of patella, tibia and tarsus; patella with small, rounded, prodistal apophysis (Fig. 2C). Tarsal claw without ventral tooth-row. *Legs*: Leg I femur length 8.1–11.0, patella 1.9–2.2, tibia 8.4–10.7; leg II femur 14.0–17.7, patella 1.9–2.5, tibia 16.0–19.0; leg III femur 7.1–9.4, patella 1.6–1.9, tibia 7.6–9.8; leg IV femur 9.0–12.2, patella 1.8–2.2, tibia 10.3–12.4. Yellow. Proximal half of femur I with few scattered dorsal denticles; remainder of legs unarmed. Tibia II with 12 pseudosegments; tibia IV with three pseudosegments. *Penis* (Fig. 2E–F): Shaft subquadrate; tendon long. Bristle groups relatively long, posterior bristle group with longest bristles reaching dorsal margin in lateral view. Glans relatively long, subrectangular in ventral view, remaining relatively deep to distal end but with dorsodistal end rounded.

#### Comments.

Females of this species are currently unknown. The holotype of *Forsteropsalis photophaga* when first examined had a parasitic mite attached to the opisthosoma (Fig. 2A). This mite is a representative of the Microtrombidiidae, a family that has not previously been recorded as parasitic on Opiliones; a more detailed description is currently being prepared by C. Taylor.

**Figure 3.**
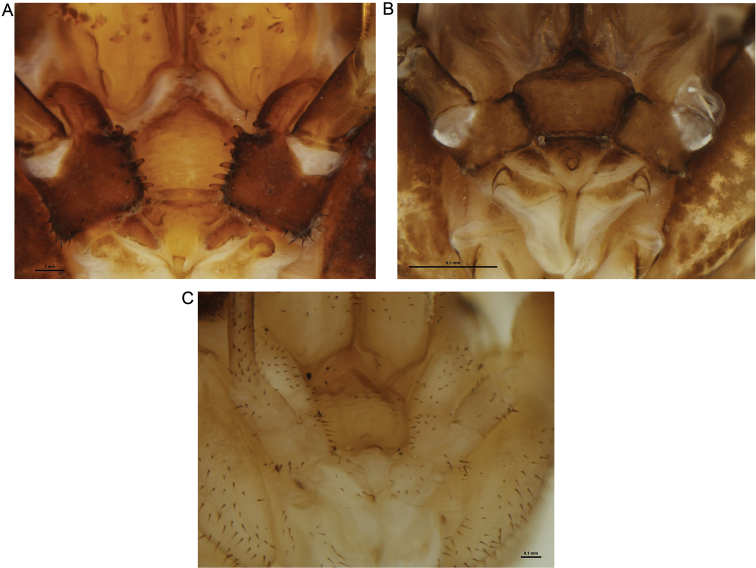
Mouthparts of selected New Zealand Neopilionidae, showing morphology of pedipalpal coxae. **A** *Forsteropsalis chiltoni*, with medial denticles **B** *Pantopsalis albipalpis*, with sclerotised medial flange overhanging cervix **C** *Mangatangi parvum*, with unarmed, simple coxae.

The genera *Pantopsalis* and *Forsteropsalis* have hitherto been regarded as well distinguished by the morphology of the cheliceral fingers (crescent-shaped in *Pantopsalis* vs bowed in *Forsteropsalis*), pedipalpal patellar apophysis (hypersetose and rounded in *Pantopsalis*, sparsely setose and triangular in *Forsteropsalis*) and penile bristle groups (shorter in *Pantopsalis* than in *Forsteropsalis*) (Taylor 2004, 2011). The current species blurs this distinction: in its hypersetose and rounded pedipalpal apophysis it resembles *Pantopsalis*, but its elongate cheliceral fingers and penile bristle groups are more characteristic of *Forsteropsalis*. It also possesses an array of denticles on the medial side of the pedipalpal coxa as found in *Forsteropsalis* species (Fig. 3A; Taylor 2011). We therefore assign it to the latter genus herein. A hypersetose, rounded patella is also present in the female of *Forsteropsalis grimmetti*, though the male of that species possesses a more typical *Forsteropsalis*-type patella (Taylor 2011). It is possible that the hypersetose patella is in fact a symplesiomorphy of *Pantopsalis* and *Forsteropsalis*, with *Forsteropsalis photophaga* being a basal member of the latter genus.

*Forsteropsalis photophaga* can be readily distinguished from all other Neopilionidae in New Zealand by the hypertrophied denticle rows on the second cheliceral segment. The only other neopilionid with comparable chelicerae is the major male of the Tasmanian species *Megalopsalis nigricans* (Hickman 1957). This, however, is a much smaller species, with very different genital morphology and with small ozopores unlike those of any *Forsteropsalis* species (Hickman 1957, Taylor 2013).

## Supplementary Material

XML Treatment for Forsteropsalis bona

XML Treatment for Forsteropsalis fabulosa

XML Treatment for Forsteropsalis photophaga
